# Nomogram based on multimodal echocardiography for assessing the evolution of diabetic cardiomyopathy in diabetic patients with normal cardiac function

**DOI:** 10.3389/fcvm.2022.1002509

**Published:** 2022-09-20

**Authors:** Yi Liu, Hao Lu, Yan Zhang, Mengjie Cai, Jia Guo, Xiaofen Ruan

**Affiliations:** ^1^Department of Ultrasonography, Shuguang Hospital Affiliated to Shanghai University of Traditional Chinese Medicine, Shanghai, China; ^2^Department of Endocrinology, Shuguang Hospital Affiliated to Shanghai University of Traditional Chinese Medicine, Shanghai, China; ^3^Department of Cardiovascular Medicine, Shuguang Hospital Affiliated to Shanghai University of Traditional Chinese Medicine, Shanghai, China

**Keywords:** diabetic cardiomyopathy, type 2 diabetes mellitus, nomogram, left ventricular diastolic dysfunction, myocardial contrast echocardiography, speckle tracking echocardiography, lifestyle intervention

## Abstract

**Background:**

Diabetic cardiomyopathy (DCM) remains asymptomatic for many years until progression to asymptomatic left ventricular diastolic dysfunction (ALVDD), a subclinical cardiac abnormality present in early-stage DCM. Because LV function in patients with type 2 diabetes mellitus (T2DM) may be subtly altered long before the onset of ALVDD, quantitative assessment of the risk of progression to early-stage DCM in T2DM patients with normal hearts is critical for delaying or even reversing DCM.

**Objective:**

This study aimed to establish a nomogram with the aid of DCM characteristics revealed by multimodal echocardiography to assess the likelihood of the progression to early-stage DCM in T2DM patients with normal cardiac function.

**Methods:**

Of the 423 T2DM patients enrolled, 302 were included in the training cohort and 121 in the validation cohort. The clinical characteristics, biochemical data, and multimodal echocardiographic parameters were collected. In the training cohort, the screened correlates of ALVDD were utilized to develop a nomogram for estimating the risk coefficient for early-stage DCM. This model was validated both in the training and validation cohorts.

**Results:**

ALVDD was independently correlated with the number of comorbidities [with one comorbidity: odds ratio (OR) = 3.009; with two comorbidities: OR = 4.026], HbA1c (OR = 1.773), myocardial blood flow (OR = 0.841), and global longitudinal strain (OR = 0.856) (all *P* < 0.05). They constituted a nomogram to visualize the likelihood of DCM development in T2DM patients with normal cardiac function. The model was validated to present strong discrimination and calibration, and obtained clinical net benefits both in the training and validation cohorts.

**Conclusion:**

We constructed and validated a nomogram to estimate the likelihood of developing early-stage DCM in T2DM patients with normal cardiac function. The alteration of the nomogram-predicted risk coefficient is expected to be proposed as a therapeutic target to slow or stop DCM progression.

## Introduction

Diabetes multiplies the risk of heart disease, and more than half of people with type 2 diabetes mellitus (T2DM) develop coronary heart disease and/or hypertension (1). However, early diabetes-related heart disease may involve only aberrant myocardial function, which is defined as “diabetic cardiomyopathy (DCM).” It causes systolic, diastolic dysfunction or both, ultimately leading to congestive heart failure (CHF), which is independent of coronary and valvular complications, hypertension, or other established HF etiologies (2, 3). The definite diagnosis of DCM remains challenging due to its unclear molecular mechanisms and frequent associated comorbidities, and it remains asymptomatic for many years (4). Asymptomatic left ventricular diastolic dysfunction (ALVDD) (5), defined as the presence of diastolic abnormalities and normal left ventricular ejection fraction (LVEF) in the absence of HF symptoms, has been described as the initial functional alteration in the course of DCM. It is the consequence of a series of cardiac insulin resistance and metabolic alteration, and precedes the development of systolic dysfunction and HF (6–8).

When combined with other comorbidities (e.g., hypertension, obesity) leading to the diagnosis of metabolic syndrome (MS), early-stage DCM may rapidly deteriorate into an advanced pathological state of cardiomyopathy, increasing the risk of cardiac dysfunction in these populations (9). Indeed, since MS is a clustering of hyperglycemia/insulin resistance, obesity, and hypertension, it places patients at a significant risk of developing cardiovascular disease. Therefore, the key is to prevent the DCM progression and effectively control the central features of MS in T2DM patients. Considering that the myocardial function in T2DM patients may have been changing subtly for a long time prior to the onset of ALVDD (10), quantitatively assessing the risk of progression to ALVDD in T2DM patients with normal hearts will be more beneficial for disease control. There is a growing consensus that the earlier DCM is intervened with proper diet and exercise, the more effectively its progression may be delayed or even reversed (11–13). Thus, evaluating the DCM evolution in T2DM patients with normal cardiac function is crucial to delay or even reverse the progression to ALVDD.

Currently, several advanced imaging techniques have been shown to overcome the difficulty of detecting subtle diastolic and systolic dysfunctions with standard echocardiography by quantifying altered myocardial microcirculatory perfusion and strain for earlier DCM detection (14–16). In this sense, multimodal echocardiography including tissue Doppler imaging, myocardial contrast echocardiography (MCE), and speckle tracking echocardiography (STE), has been regarded as a non-invasive approach to identify cardiac dysfunction in T2DM patients (17–20), and help assess the disease progression and therapy effectiveness (21). However, for T2DM patients with normal hearts, relevant studies focusing on the risk stratification of DCM evolution are limited, although there is data on lifestyle interventions to reverse cardiovascular dysfunction, including weight loss, increased aerobic exercise capacity, etc. (22–25).

With this background, we aimed to establish a nomogram to assess the DCM evolution in T2DM patients with normal cardiac function based on the DCM characteristics revealed by multimodal echocardiography. The alteration of the nomogram-predicted risk coefficient may help delay the progression of DCM effectively.

## Materials and methods

This was a retrospective study and it complied with the principles stated in the declaration of Helsinki. It was approved by the Institutional Review Board of Shuguang Hospital Affiliated Shanghai University of Traditional Chinese Medicine (2020-901-110-01). All patients supplied informed consents.

### Study population

Using medical records, we reviewed 653 T2DM patients receiving multimodal echocardiography at Shuguang Hospital Affiliated Shanghai University of Traditional Chinese Medicine between April 2019 and March 2022. Diabetes is diagnosed using the American Diabetes Association criteria (26). Cardiological examinations were systematically performed in all patients. The following inclusion criteria were met by all patients: (1) age between 35 and 60 years, (2) LVEF ≥ 55%, and (3) no indication of coronary artery disease, valvular disease, HF, or arrhythmia based on history, electrocardiogram, echocardiography, and metabolic exercise stress test, according the definition of DCM, a clinical form of CHF with normal coronary arteries and no other etiologies for CHF (27). Patients with left ventricular hypertrophy were excluded as we focused primarily on clinical features associated with early-stage DCM. In addition, patients with severe diabetic complications such as nephropathy or diabetic foot, uncontrolled hypertension, congenital heart disease, stroke, malignancy, cirrhosis, and significant renal impairment were also excluded.

**Figure 1:**
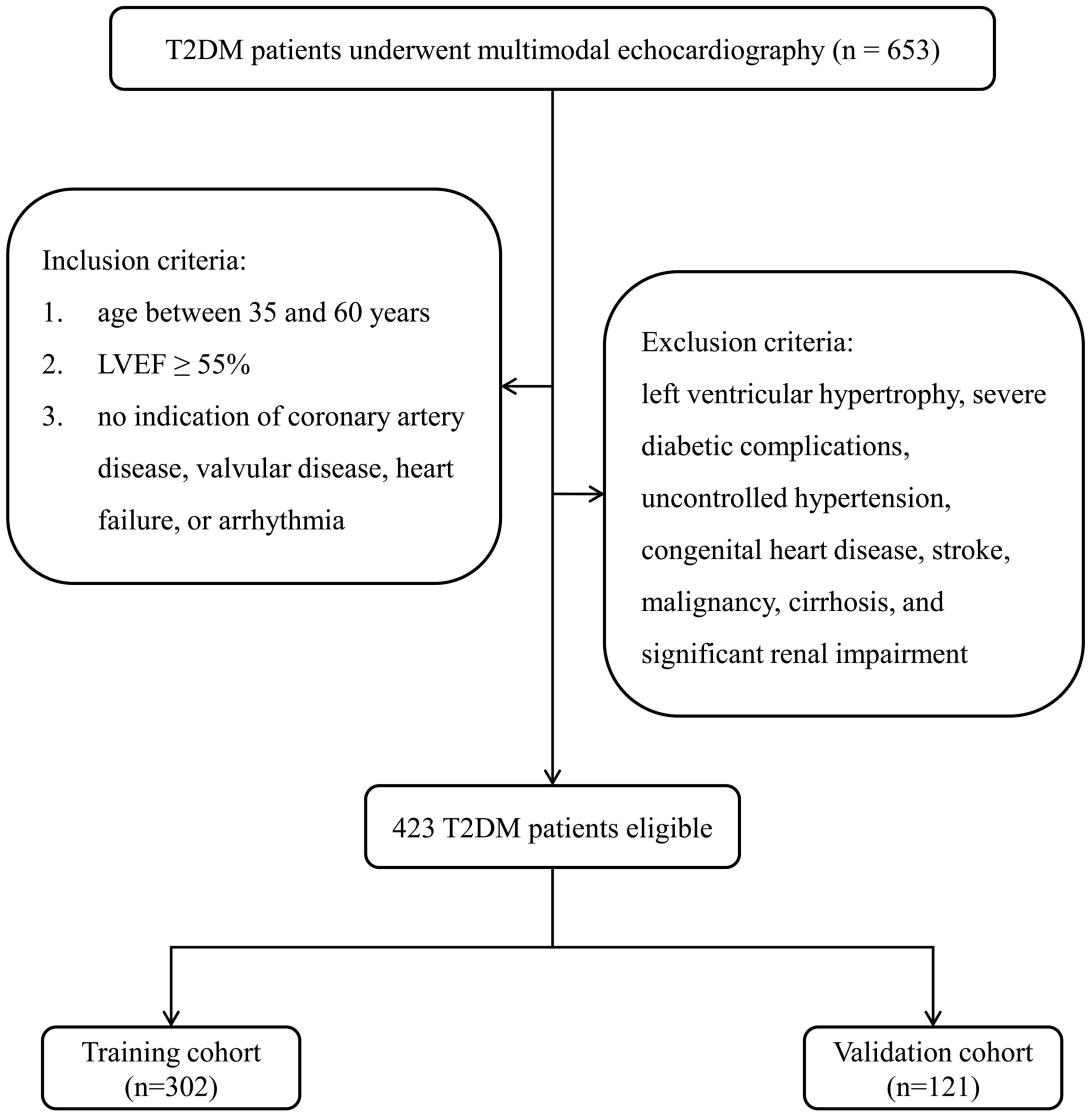
Flow diagram for the construction of training and validation cohorts. T2DM, type 2 diabetes mellitus; LVEF, left ventricular ejection fraction.

Ultimately, 423 strictly screened medical records of T2DM patients were eligible for the analysis. In order to independently validate the established nomogram, patients from April 2019 to March 2021 were included in a training cohort (*n* = 302) and those from April 2021 to March 2022 were assigned to a validation cohort (*n* = 121). Figure 1 depicts a flow diagram exhibiting patient selection and the construction of training and validation cohorts.

### Collection of clinical characteristics and biochemical data

Data of clinical characteristics were reviewed from medical records, including diabetic duration, body mass index (BMI), body surface area (BSA) [calculated as 0.0061 × height (cm) + 0.0128 × weight (kg) −0.1529], blood pressure, smoking and drinking status, comorbidities (hypertension and fatty liver), and medication intake. Laboratory tests included fasting blood glucose (FBG), glycated hemoglobin (HbA1c), total cholesterol (TC), triglycerides (TG), high-density lipoprotein cholesterol (HDL), and low-density lipoprotein cholesterol (LDL), according to standard procedures.

### Echocardiographic acquisition and analysis

A comprehensive echocardiographic examination was performed for all patients by utilizing EPIQ 7C ultrasound system (Philips, Andover, MA). Offline image analysis was performed using Philips QLAB quantification software.

Routine cardiac parameters included left ventricular end diastolic dimension (LVDd), left ventricular end systolic dimension (LVDs), posterior wall thickness (PWT), and interventricular septum thickness (IVST). Left atrial volume index (LAVI) was calculated using the biplane area-length method (28). LVEF was measured by the Simpson's biplane volumetric method. In color flow Doppler and tissue Doppler, E/A ratio (E wave, early ventricular filling; A wave, late ventricular filling), isovolumetric relaxation time (IVRT), deceleration time (DT), tricuspid regurgitation peak velocity (TRPV), and E/e' ratio (e', average velocity measured at the septal and lateral sides of mitral annulus) were obtained.

The procedure of MCE has been previously reported (19) and is briefly described here. After the myocardium was filled with the Sonovue contrast agent (Bracco, Milan, Italy), a burst of high-Mechanical Index (1.35) was triggered to deplete contrast microbubbles within the myocardium. Then, the replenishment of myocardial microbubbles was recorded from the apical four-, two-, and three-chamber view over 15 cardiac cycles, which were stored for offline analysis. The replenishment curve of the region of interest (ROI) in each apical segment was obtained. The global *A*, β, and *A* × β, where *A* represents myocardial blood volume, β represents myocardial perfusion (MP) velocity, and *A* × β represents myocardial blood flow (MBF), were determined by averaging all ROIs.

Two-dimensional STE analysis was performed offline to assess the LV function. LV systolic function was assessed by measuring LV peak systolic global longitudinal strain (GLS) and global radial strain (GRS). GLS was average from apical long-axis, two-chamber, and four-chamber views, while GRS from apical, mid-ventricular, and basal short-axis views.

### Study outcome

Given the high prevalence of DCM in T2DM patients, screening for its presence at the earliest stage of development would be appropriate to prevent the progression to CHF (29). However, due to the difficulty in definite DCM diagnosis, this study defined ALVDD, the initial functional alteration of early-stage DCM (30), as the study outcome to investigate the differences in asymptomatic T2DM patients with ALVDD and with normal cardiac function. According the recommendations for ALVDD evaluation, ALVDD was diagnosed when ≥3 of the following criteria were met: septal e' < 7 cm/s or lateral e' < 10 cm/s, average E/e' > 14, TRPV > 2.8 m/s, and LAVI > 34 ml/m^2^ (31).

### Nomogram establishment

In the training cohort, the association of each characteristic with the diagnosis of ALVDD was assessed in the least absolute shrinkage and selection operator (LASSO) based algorithm. Then all initially screened variables were included in a multivariate Logistic regression to identify the risk factors for ALVDD. The final step was to build a nomogram for estimating the risk coefficient for early-stage DCM. The performance of the model was first internally evaluated in the training cohort and then externally verified by fitting it to the validation cohort with the same parameter estimates as the training cohort.

### Statistical analysis

Continuous variables were expressed as mean ± standard deviation or median (interquartile range) depending on whether they exhibited normal distribution. The variables which considered statistically significant (*P* < 0.05) in the LASSO model were included in the multivariate Logistic regression analysis to calculate the odds ratio (OR) and 95% confidence interval (CI) for each independent correlate of ALVDD. The nomogram was constructed based on these correlates to predict the risk of DCM in T2DM patients with normal cardiac function. In the internal model validation, bootstraps (1,000 times) analyses were employed to prevent the possible overfitting deviation. The discrimination and calibration were assessed by an area under the receiver operating characteristic (ROC) curve (AUC) and a calibration curve with a Hosmer-Lemeshow (HL) test. A decision curve analysis (DCA) was conducted to quantify the net benefits at different risk threshold probabilities. For external validation, the discrimination, calibration, and net benefit were evaluated by applying the trained model to the validation cohort without retraining. All statistical analyses were carried out using SPSS software (Version 22.0) and R package (Version 4.1.3).

## Results

### Patient characteristics

ALVDD was diagnosed in 138 of the 423 eligible T2DM patients (32.6%). In this study, 302 patients were assigned to the training cohort to build the prediction model, and 121 were in the validation cohort to test the model performance. The comparisons of clinical information and laboratory tests between the training and validation cohorts are summarized in Table 1, while Table 2 gives the comparisons of routine echocardiographic, MCE, and STE parameters. The anti-diabetic medications in normoglycemic vs. hyperglycmeic patients are listed in Appendix in Supplementary material. No significant differences among ALVDD incidence, clinical information, laboratory tests, and multimodal echocardiographic parameters were observed between the two cohorts (all *P* > 0.05), implying that the validation cohort was applicable for the external validation of the model built in the training cohort.

**Table 1:** Comparisons of clinical information and laboratory tests in eligible T2DM patients between the training and validation cohorts.

**Item**	**Training cohort**	**Validation**	***P*-value**
	**cohort**	**cohort**	
	**(*n* = 302)**	**(*n* = 121)**	
**Clinical information**	
Age, year	47.36 ± 6.14	46.39 ± 5.38	0.129*
Diabetic duration, year	4 (3–5)	3 (3–5)	0.302^$^
**Gender**, ***n*** **(%)**	
Male	177 (58.6%)	67 (55.4%)	0.543^#^
Female	125 (41.4%)	54 (44.6%)	
BMI, kg/m^2^	27.5 (25.2–29.6)	26.7 (24.1–28.5)	0.134^$^
BSA, m^2^	1.66 ± 0.11	1.68 ± 0.13	0.110*
SBP, mmHg	127 (121–133)	125 (118–131)	0.386^$^
DBP, mmHg	80 (74–85)	82 (77–89)	0.206^$^
Smoking, *n* (%)	102 (33.8%)	37 (30.6%)	0.527^#^
Drinking, *n* (%)	138 (45.7%)	45 (37.2%)	0.111^#^
**Comorbidities**, ***n*** **(%)**	
None	48 (15.9%)	17 (14.1%)	0.635^#^
Hypertension	173 (57.3%)	65 (53.7%)	0.504^#^
Fatty liver	153 (50.7%)	55 (45.5%)	0.333^#^
**Medication**, ***n*** **(%)**	
Metformin, *n* (%)	235 (77.8%)	98 (80.9%)	0.471^#^
DPP4 inhibitors, *n* (%)	170 (56.3%)	61 (50.4%)	0.272^#^
SGLT-2 inhibitors, *n* (%)	115 (38.1%)	41 (33.9%)	0.419^#^
Insulin, *n* (%)	57 (18.9%)	28 (23.1%)	0.322^#^
ACEI/ARBs, *n* (%)	147 (48.7%)	60 (49.6%)	0.865^#^
Calcium blockers, *n* (%)	87 (28.8%)	33 (27.3%)	0.752^#^
Diuretics, *n* (%)	27 (8.9%)	12 (9.9%)	0.754^#^
Statins, *n* (%)	187 (61.9%)	67 (55.4%)	0.214^#^
**Laboratory tests**	
FBG, mmol/L	9.25 ± 2.46	9.01 ± 2.15	0.348*
HbA1c, %	6.4 (4.9–7.5)	6.8 (5.1–7.7)	0.412^$^
TC, mmol/L	2.14 ± 0.76	2.23 ± 0.53	0.234*
TG, mmol/L	4.08 ± 0.76	3.99 ± 0.65	0.253*
HDL, mmol/L	1.05 ± 0.19	1.02 ± 0.13	0.112*
LDL, mmol/L	2.29 ± 0.53	2.39 ± 0.65	0.102*

^*^For independent sample t-test, ^#^for chi-square test, and ^$^for Mann-Whitney U-test. BMI, body mass index; BSA, body surface area; SBP, systolic blood pressure; DBP, diastolic blood pressure; SGLT-2, sodium–glucose co-transporter-2; DPP4, dipeptidyl peptidase-4; ACEI, angiotensin-converting enzyme inhibitors; ARBs, angiotensin receptor blockers; FBG, fasting blood glucose; HbA1c, glycated hemoglobin; TC, total cholesterol; TG, triglycerides; HDL, high-density lipoprotein cholesterol; LDL, low-density lipoprotein cholesterol.

**Table 2:** Comparison of multimodal echocardiographic parameters (rountine echocardiography, MCE, and STE) between the training and validation cohorts.

**Item**	**Training cohort** ** (*n* = 302)**	**Validation cohort** ** (*n* = 121)**	***P*-value**
Diagnosis of ALVDD, *n* (%)	105 (34.8%)	33 (27.3%)	0.137^#^
**Routine echocardiographic parameters**			
LVDd, mm	45.4 ± 5.3	46.3 ± 5.2	0.113*
LVDs, mm	27.3 ± 4.6	28.0 ± 4.9	0.166*
IVST, mm	9.3 ± 1.2	9.1 ± 1.3	0.131*
PWT, mm	9.3 ± 1.0	9.2 ± 1.1	0.367*
LVEF, %	63.2 ± 6.1	62.1 ± 5.8	0.109*
LAVI, ml/m^2^	29.6 (24.4–35.8)	32.3 (23.3–37.4)	0.153^$^
A velocity, cm/s	77 (68–86)	80 (70–91)	0.342^$^
E velocity, cm/s	91 (79–101)	91 (80–103)	0.241^$^
E/A ratio	0.83 (0.71–0.98)	0.81 (0.67–0.96)	0.283^$^
DT, ms	231.0 ± 46.7	233.2 ± 45.7	0.660*
IVRT, ms	88.9 ± 12.2	91.5 ± 16.4	0.075*
TRPV, m/s	2.53 ± 0.36	2.61 ± 0.43	0.146*
Septal e', cm/s	8.04 ± 1.14	8.32 ± 1.25	0.127*
Lateral e', cm/s	8.94 ± 1.93	9.16 ± 1.77	0.279*
Average E/e'	7.92 ± 1.68	8.25 ± 1.53	0.162*
**MCE parameters**			
*A*, dB	21.7 (20.6–23.2)	22.4 (21.1–23.8)	0.373^$^
β, dB/s	1.1 (0.9–1.2)	1.2 (1.0–1.3)	0.264^$^
MBF, dB^2^/s	22.2 (17.3–29.1)	24.1 (18.1–30.3)	0.163^$^
**STE parameters**			
GLS, %	−19.6 ± 3.1	−18.7 ± 3.2	0.373*
GRS, %	44.9 ± 8.1	46.3 ± 7.5	0.102*

^*^For independent sample t-test, ^#^for chi-square test, and ^$^for Mann-Whitney U-test. ALVDD, asymptomatic left ventricular diastolic dysfunction; LVDd, left ventricular end-diastolic dimension; LVDs, left ventricular end-systolic dimension; IVST, inter-ventricular septum thickness; PWT, posterior wall thickness; LVEF, left ventricular ejection fraction; LAVI, left atrial volume index; A, peak velocity in late diastole; E, peak velocity in early diastole; DT, deceleration time; IVRT, isovolumetric relaxation time; TRPV, tricuspid regurgitation peak velocity; e', early diastolic mitral annular velocity; MCE, myocardial contrast echocardiography; *A*, myocardial blood volume; β, myocardial perfusion velocity; MBF, myocardial blood flow; STE, speckle tracking echocardiography; GLS, global longitudinal strain; GRS, global radial strain.

### Predictor selection based on LASSO model

**Figure 2:**
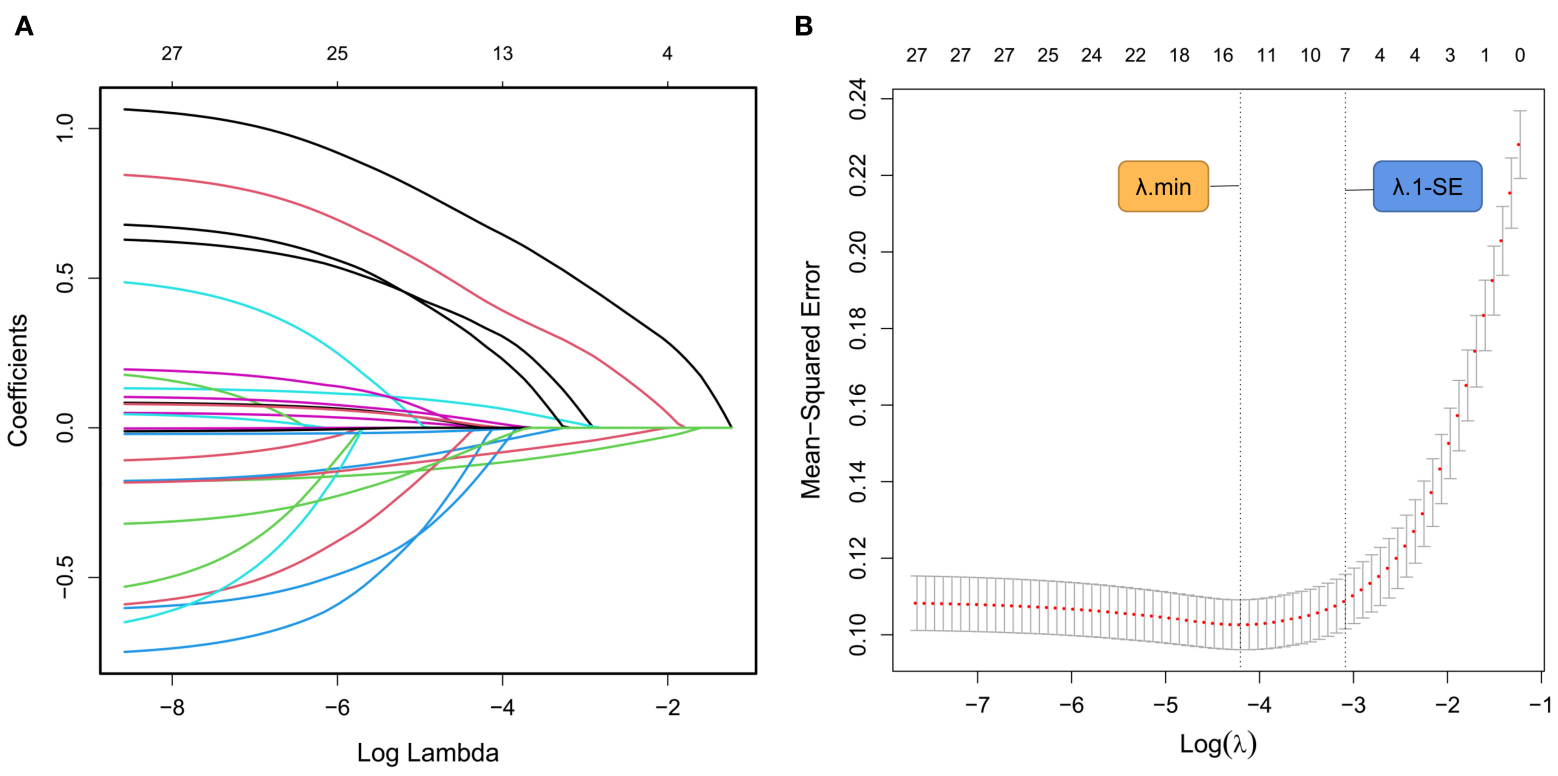
ALVDD-related predictors selected by the LASSO model. **(A)** Displays the LASSO coefficient profile for each candidate predictor. Each colored curve depicts the trajectory of each variable coefficient. The λ is identified by 10-fold cross-validation. **(B)** Draws the tuning λ selection in the LASSO regression based on the minimum criteria (λ.min, left dashed line) and the 1-standard error for the minimum criteria (λ.1-SE, right dashed line). In the present study, seven variables (diabetic duration, BMI, number of comorbidities, HbA1c, MBF, GLS, and GRS) are chosen by determining the optimal λ value with λ.1-SE. ALVDD, asymptomatic left ventricular diastolic dysfunction; LASSO, least absolute shrinkage and selection operator; λ, penalty regularization parameter; BMI, body mass index; HbA1c, glycated hemoglobin; MBF, myocardial blood flow; GLS, global longitudinal strain; GRS, global radial strain.

In the training cohort, the LASSO model included all available clinical indicators as candidate variables associated with ALVDD except those used for ALVDD diagnosis, including septal e', lateral e', average E/e', TRPV, and LAVI. Seven variables were chosen by determining the best penalty regularization parameter (λ) with the 1-standard error for the minimum criteria, including diabetic duration, BMI, number of comorbidities, HbA1c, MBF, GLS, and GRS (Figure 2).

### Independent correlates of ALVDD

**Figure 3:**
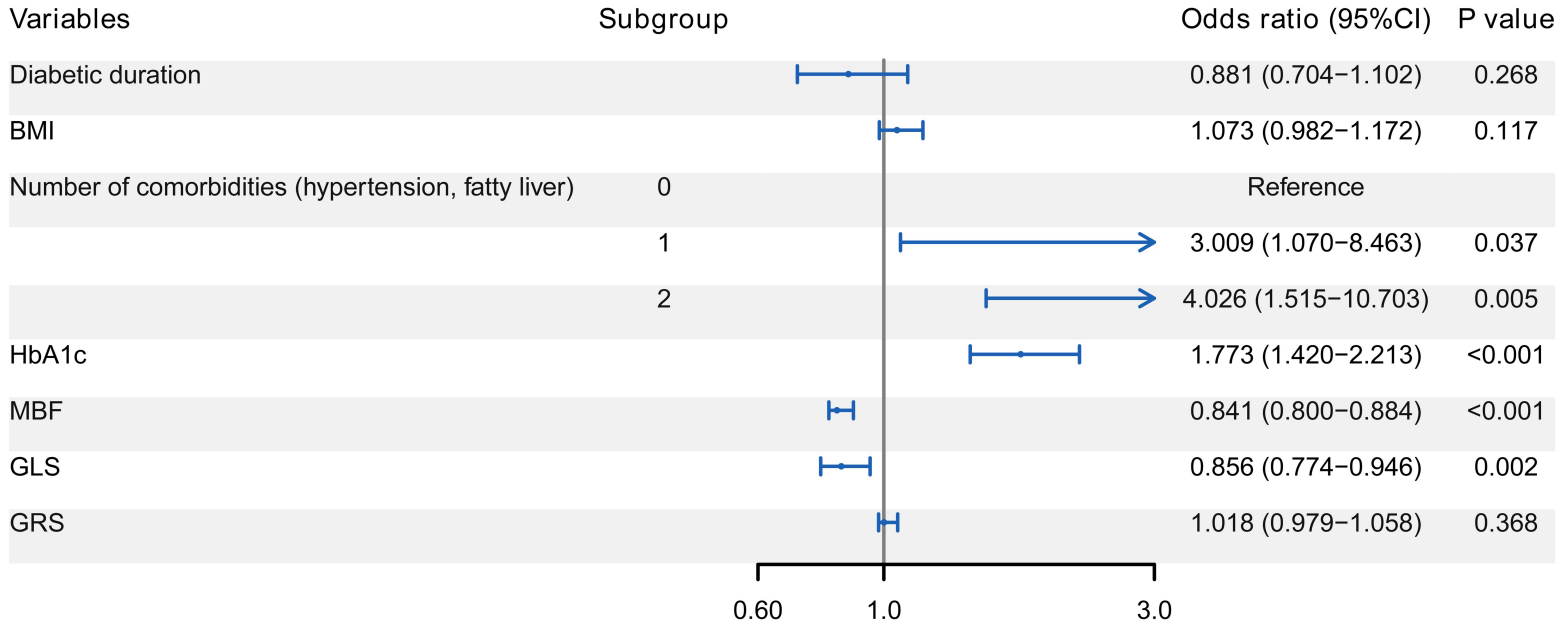
Forest plot of multivariate Logistic regression analysis for the correlates of ALVDD. It reveals that ALVDD is independently correlated with the number of comorbidities, HbA1c, MBF, and GLS (all *P* < 0.05). ALVDD, asymptomatic left ventricular diastolic dysfunction; T2DM, type 2 diabetes mellitus; HbA1c, glycated hemoglobin; MBF, myocardial blood flow; GLS, global longitudinal strain.

The seven variables further entered the multivariate Logistic regression analysis to determine the independent correlates of ALVDD. Figure 3 shows that ALVDD was independently correlated with the number of comorbidities, HbA1c, MBF, and GLS (all *P* < 0.05). Patients with 1 or 2 comorbidities were more likely to have ALVDD, with ORs of 3.009 and 4.026, respectively. Increased HbA1c and decreased MBF as well as GLS were associated with ALVDD, with ORs of 1.773, 0.841, and 0.856, respectively.

### Model development

**Figure 4:**
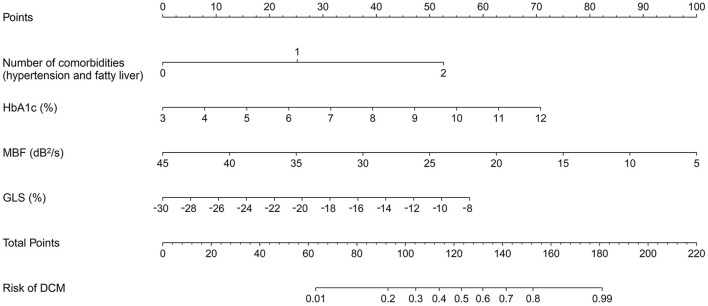
Nomogram for estimating the chance of developing DCM in T2DM patients with normal heart. It is developed with the number of comorbidities, HbA1c, MBF, and GLS with weights equal to the OR values. DCM, diabetic cardiomyopathy; T2DM, type 2 diabetes mellitus; HbA1c, glycated hemoglobin; MBF, myocardial blood flow; GLS, global longitudinal strain; OR, odds ratio.

To visualize the likelihood of DCM progression in T2DM patients with normal cardiac function, a nomogram was developed based on the variables determined by the multivariate analysis. With weights corresponding to the OR values, the points of each variable were added to determine the likelihood of DCM progression, as shown in Figure 4. The total point matched the risk of DCM on the bottom axis. For example, in a 48-year-old T2DM patient with hypertension and normal cardiac function, his HbA1c, MBF, and GLS was 7%, 22dB^2^/s and −20%, respectively. The corresponding scores for these features in the nomogram were: 25 points for the number of comorbidities, 31 points for HbA1c, 57 points for MBF, and 25 points for GLS. His overall score was around 138, meaning that he had a roughly 65% chance of progressing to ALVDD.

### Model validation

**Figure 5:**
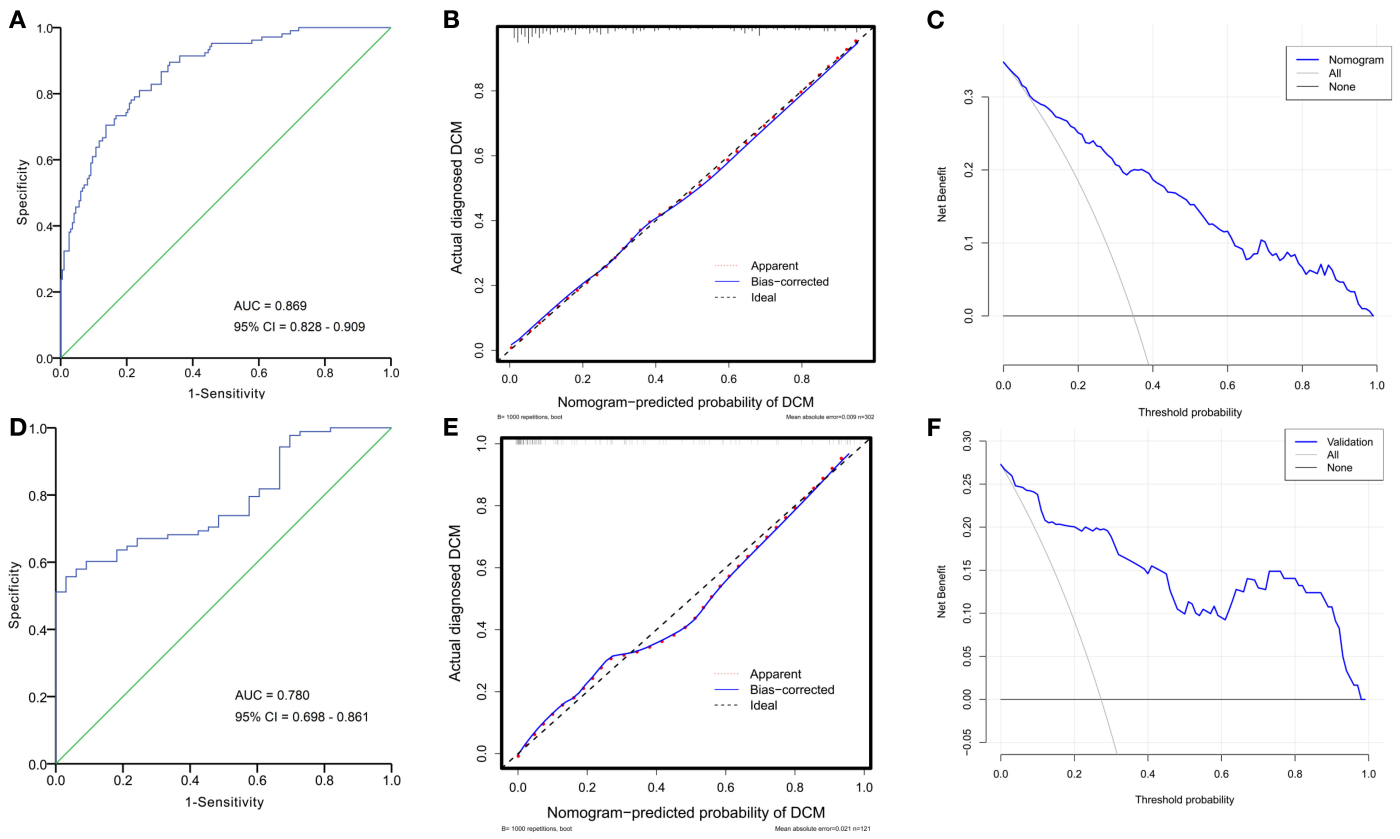
ROC curves, calibration plots, and DCA curves for evaluating the performance of the established nomogram in the training and validation cohorts. In the training cohort, the AUC of ROC curve in **(A)** is 0.869 (95% CI: 0.828–0.909), suggesting good discrimination. The calibration plot in **(B)** indicates good agreement between predicted and actual probabilities of DCM. The DCA curve in **(C)** reveals a clinical net benefit. Implying that the nomogram will add significantly more benefit than either the treat-all scheme or the treat-none scheme. In the validation cohort, the nomogram still shows good discrimination (AUC = 0.780, 95% CI: 0.698–0.861) **(D)**, calibration **(E)**, and clinical utility **(F)**. ROC, receiver operating characteristic; AUC, area under the curve; DCA, decision curve analysis; CI, confidence interval.

The performance of the developed model was first evaluated based on the training cohort. After 1,000 bootstrapping, its stability was evaluated in order to correct the overfitting deviation. The discrimination of the nomogram was tested with the ROC curve, as indicated in Figure 5A. The AUC was 0.869 (95% CI: 0.828–0.909), indicating good discrimination (AUC > 0.75). The calibration curve (Figure 5B) revealed no significant difference between the predicted and actual probabilities of ALVDD, and HL test yielded a nonsignificant value (χ^2^ = 4.274, *P* = 0.832), which suggested that the model was well-calibrated. The DCA plot in Figure 5C showed that the use of the nomogram to predict the risk of DCM would add a net benefit. Then, external validations were applied to test the usefulness of the nomogram. After the original model applied to the validation cohort, the nomogram still showed good discrimination (AUC = 0.780, 95% CI: 0.698–0.861) (Figure 5D) and calibration assessed by the HL test (χ^2^ = 6.294, *P* = 0.627) (Figure 5E). The DCA plot in Figure 5F showed that the application of this nomogram in the validation cohort to predict the risk of ALVDD progression would generate clinical net benefits, indicating a good potential for clinical utility.

## Discussion

Since DCM-induced CHF is one of the leading causes of premature mortality in T2DM patients, accurate evaluation of DCM evolution and appropriate treatment are crucial for reducing cardiovascular complications in diabetic patients. In the present study, we developed and validated a nomogram, including the number of comorbidities, HbA1c, MBF, and GLS, to assess the likelihood of developing early-stage DCM in T2DM patients with normal cardiac function. It adds important new insights into the risk stratification of DCM evolution, as our observation implied that the alteration of multimodal echocardiographic parameters might be useful in determining myocardial dysfunction in the preclinical stage of DCM. To our knowledge, this study is the first to construct a nomogram for assessing the DCM evolution in T2DM patients with normal cardiac function. If further verified, the nomogram-predicted risk coefficient may be proposed as a therapeutic target, allowing for the prompt implementation of tailored therapy options to slow or stop the DCM progression.

### Clinical significance of the nomogram

DCM has been reported to be reversible in its early and even preclinical stages (32, 33). However, intensive glycemic control alone did not reduce the risk of DCM progression, according to a meta-analysis of data from 8 randomized trials of 37,229 T2DM patients (34). Emerging evidences suggest that glycemic control in addition to lifestyle interventions, including low-calorie diets, as well as aerobic and resistance training etc., can reverse diabetes and even DCM (35–37). More importantly, these substantial benefits persisted even when the intensity of exercise training was insufficient for weight loss (38). The European association of preventive cardiology also supports that personalized exercise training enhances cardiovascular and metabolic function in T2DM patients (39). Although further research is needed to improve the structural and functional disturbances of the diabetic heart through such lifestyle changes, a strong causal relationship has been demonstrated between lifestyle changes and the evolution of DCM. However, in fact, patients frequently struggle to maintain long-term lifestyle interventions due to their failure to recognize subtle changes in cardiac function, which leads to DCM progression. This requires nomograms to dynamically estimate the risk of DCM evolution, thereby improving their motivation and adherence.

### Composition of the nomogram

The components of the nomogram included the number of comorbidities, HbA1c, MBF, and GLS, which are all independent correlates of ALVDD. Diabetes, hypertension, and obesity are recognized factors associated with ALVDD. Together with atherogenic dyslipidemia, they are the central features of MS. These conditions are interrelated and share common mediators, pathways and pathophysiological mechanisms (9). MS is driven by a range of metabolic abnormalities and is a recognized risk factor for cardiovascular morbidity and mortality (40–42). A recent large community-based study of 6,814 patients without initial coronary artery disease showed that increasing indices of MS tracked with increasing HF risk, further underscoring the importance of metabolic disorders in cardiovascular disease (43). Myocardial dysfunction in T2DM patients has been confirmed by numerous studies, which is consistent with the findings of our study (44–46). It should be accurately assessed by multimodal echocardiography including MCE and STE to detect impaired MP and LV dyssynchrony during diabetes progression. MCE is developed for quantitatively evaluating myocardial microcirculation and coronary microvasculature in real time (47). We exclusively chose the apex to observe MP because coronary microcirculation gradually decreases from the base to the apex (48, 49), and histopathological studies have verified that the damaged MP first manifested in the apex (50). With the subtle progression of DCM, it was previously thought that systolic dysfunction usually followed diastolic dysfunction (51), but with advances in knowledge, more and more studies have discovered that isolated diastolic dysfunction is rare, often combined with subclinical systolic dysfunction (16, 52–54). Ernande et al. (55) even found that 28% of T2DM patients showed systolic strain abnormalities preceding diastolic dysfunction. Systolic dysfunction is initially apparent in the longitudinal direction, since subendocardial longitudinal fibers are more susceptible to myocardial ischemia and fibrosis (56, 57). According to our findings, ALVDD was independently associated with the GLS abnormalities found by STE, suggesting that this may be another early alteration seen in the early-stage of DCM. Recent studies demonstrate that impaired GLS in asymptomatic T2DM patients is associated with adverse cardiovascular outcomes and offers incremental prognostic value for up to a decade after revelation (58, 59). It is worth noting, however, that GLS is afterload-dependent and its value may be affected by arterial hypertension. A recent meta-analysis indicated that higher prevalence of hypertension and higher HbA1c were both main contributors to worse GLS in T2DM patients (60). Therefore, it is not necessary to distinguish whether the exacerbation of GLS is caused by DCM or hypertension. Indeed, hypertension shares similar pathogenic pathways with DCM, which accelerates the underlying process of myocardial dysfunction (61). When DCM is associated with hypertension, cardiac dysfunction is more common and more likely to become clinically apparent in diabetic patients, suggesting that myocardial damage is maximized in the presence of both diabetes and hypertension (62, 63).

### Utility of the nomogram

Only a small number of models are available for predicting the cardiovascular diseases of T2DM patients. Shi et al. (64) established a nomogram for predicting the risk of coronary heart disease after systematically analyzing the physical and biochemical data of T2DM patients in six communities, but DCM was not considered. Chen et al. (65) developed a model for predicting the risk of DCM-induced ALVDD, but no further verification was conducted due to the small sample size. In our experience, this is the first study to estimate the DCM evolution in T2DM patients with normal hearts by a nomogram. This model was verified to show favorable discrimination and calibration, as well as considerable clinical net benefits both in the training and validation cohorts. When implementing a long-term lifestyle modification, it is advantageous to dynamically assess the risk of DCM progression. In a T2DM patient with normal cardiac function as mentioned in the results section, he had a 65% risk of DCM progression. It implies that more intensive glucose control and lifestyle interventions may be encouraged. Moreover, review after 1 year is advised to ensure his risk is <50% because DCM has been observed to worsen as diabetes progresses without proper intervention (66, 67).

### Limitation

It is important to recognize some limitations. First, because this study was retrospective in nature, it was inherently subject to selection bias and some techniques, like cardiac magnetic resonance imaging and non-invasive myocardial work assessment, were not included. Besides, some anti-diabetic medications that might affect cardiac function, such as sodium–glucose co-transporter-2 (SGLT-2) inhibitors (68), had not been further investigated. A prospective observational study is planned to investigate the effects of anti-diabetic medications such as SGLT-2 inhibitors (69) and anti-remodeling treatments like angiotensin receptor-neprilysin inhibitors (70) on ameliorating cardiac remodeling and cardiac dysfunction. Second, not all T2DM patients were originally screened for coronary artery disease with coronary computed tomography angiography due to the radiation exposure. Nevertheless, the focus of this study is not on the definite diagnosis of DCM, but rather on assessing the extent of the progression of myocardial dysfunction caused by DCM. In fact, DCM often coexists and interacts with various cardiovascular risk comorbidities, and they often accelerate the worsening of myocardial dysfunction. Therefore, it is critical for accurate assessment of cardiac function changes in diabetic patients. Finally, the study was conducted in a single center even though the model verification was performed in a validation cohort. Future multicenter prospective studies will be necessary to improve the individualized risk stratification provided by our model.

## Conclusion

We developed a nomogram that included the number of comorbidities, HbA1c and multimodal echocardiographic parameters to estimate the evolution of DCM in T2DM patients with normal cardiac function. Dynamic changes in the risk coefficient of DCM progression may help improve their insufficient adherence to long-term lifestyle interventions and allow adjustment of therapeutic strategies to prevent the progression to advanced DCM.

## Data availability statement

The raw data supporting the conclusions of this article will be made available by the authors, without undue reservation.

## Ethics statement

The studies involving human participants were reviewed and approved by ethical approval for the study was obtained from the Institutional Review Board of Shuguang Hospital Affiliated Shanghai University of Traditional Chinese Medicine (2020-901-110-01). The patients/participants provided their written informed consent to participate in this study.

## Author contributions

YL, HL, YZ, JG, and XR: study design and manuscript revision. YL, HL, YZ, and MC: data collection, analysis, and statistics. JG and XR: supervision. YL, HL, and YZ: manuscript writing. All authors approval of the manuscript.

## Funding

This study was supported by the National Natural Science Foundation of China (82174152), Shanghai Shenkang development research project (SHDC12018X29), and National Natural Science Foundation of China (82074381).

## Conflict of interest

The authors declare that the research was conducted in the absence of any commercial or financial relationships that could be construed as a potential conflict of interest.

## Publisher's note

All claims expressed in this article are solely those of the authors and do not necessarily represent those of their affiliated organizations, or those of the publisher, the editors and the reviewers. Any product that may be evaluated in this article, or claim that may be made by its manufacturer, is not guaranteed or endorsed by the publisher.
