# Supplementary material for: Nomogram based on multimodal echocardiography for assessing the evolution of diabetic cardiomyopathy in diabetic patients with normal cardiac function
Source: Front Cardiovasc Med. 2022 Sep 20;9:1002509. doi: 10.3389/fcvm.2022.1002509 (PMC9530038; doi:10.3389/fcvm.2022.1002509)
Supplement: Supplementary file 1 [file Table_1.docx]

**Supplementary**

**Table 1**. Comparison of anti-diabetic medications in normoglycemic vs. hyperglycmeic patients

| **Item** | | **Normoglycemic patients (n=337)** | **Hyperglycmeic patients (n=86)** | ***P* value** |
| --- | --- | --- | --- | --- |
| Anti-diabetic medication, n(%) | Metformin, n(%) | 265 (78.6%) | 68 (79.1%) | 0.930^#^ |
|  | DPP4 inhibitors, n(%) | 180 (53.4%) | 51 (59.3%) | 0.327^#^ |
|  | SGLT-2 inhibitors, n(%) | 108 (32.1%) | 48 (55.8%) | <0.001^#^ |
|  | Insulin, n(%) | 48 (14.2%) | 37 (43.0%) | <0.001^#^ |

^#^for chi-square test. SGLT-2: sodium–glucose co-transporter-2, DPP4: dipeptidyl peptidase-4.
